# Identification of microRNAs in developing wheat grain that are potentially involved in regulating grain characteristics and the response to nitrogen levels

**DOI:** 10.1186/s12870-020-2296-7

**Published:** 2020-02-27

**Authors:** Gege Hou, Chenyang Du, Honghuan Gao, Sujun Liu, Wan Sun, Hongfang Lu, Juan Kang, Yingxin Xie, Dongyun Ma, Chenyang Wang

**Affiliations:** 1 College of Agronomy/National Engineering Research Center for Wheat, Henan Agricultural University, Zhengzhou, 450002 China; 2 The National Key Laboratory of Wheat and Maize Crop Science, Henan Agricultural University, Zhengzhou, 450002 China

**Keywords:** Nitrogen application level, Differentially expressed miRNAs, Grain development, Wheat

## Abstract

**Background:**

MicroRNAs (miRNAs) play crucial roles in the regulation of plant development and growth, but little information is available concerning their roles during grain development under different nitrogen (N) application levels. Our objective was to identify miRNAs related to the regulation of grain characteristics and the response to different N fertilizer conditions.

**Results:**

A total of 79 miRNAs (46 known and 33 novel miRNAs) were identified that showed significant differential expression during grain development under both high nitrogen (HN) and low nitrogen (LN) treatments. The miRNAs that were significantly upregulated early in grain development target genes involved mainly in cell differentiation, auxin-activated signaling, and transcription, which may be associated with grain size; miRNAs abundant in the middle and later stages target genes mainly involved in carbohydrate and nitrogen metabolism, transport, and kinase activity and may be associated with grain filling. Additionally, we identified 50 miRNAs (22 known and 28 novel miRNAs), of which 11, 9, and 39 were differentially expressed between the HN and LN libraries at 7, 17, and 27 days after anthesis (DAA). The miRNAs that were differentially expressed in response to nitrogen conditions target genes involved mainly in carbohydrate and nitrogen metabolism, the defense response, and transport as well as genes that encode ubiquitin ligase. Only one novel miRNA (PC-5p-2614_215) was significantly upregulated in response to LN treatment at all three stages, and 21 miRNAs showed significant differential expression between HN and LN conditions only at 27 DAA. We therefore propose a model for target gene regulation by miRNAs during grain development with N-responsive patterns.

**Conclusions:**

The potential targets of the identified miRNAs are related to various biological processes, such as carbohydrate/nitrogen metabolism, transcription, cellular differentiation, transport, and defense. Our results indicate that miRNA-mediated networks, via posttranscriptional regulation, play crucial roles in grain development and the N response, which determine wheat grain weight and quality. Our study provides useful information for future research of regulatory mechanisms that focus on improving grain yield and quality.

## Background

As one of the largest and most widely planted food crop species in the world, wheat provides approximately 20% of the required human calorie intake [1]. With the rapid increase in the global population, increasing wheat yields is a major goal to meet future food demands [2]. Additionally, with increasing economic development, improving wheat quality, including increasing the protein content, is another important objective for wheat production. Conventional breeding approaches, which depend on the manipulation of genetic variation, have been successful at producing wheat cultivars with improved grain yield and quality; however, understanding the molecular mechanisms that regulate wheat development and grain quality would benefit further wheat improvement programs via modern genetic engineering technology [3].

Small RNAs (sRNAs) are ubiquitous components of plant transcriptomes; they have been found to regulate cellular metabolism, differentiation and growth and are involved in defense against viruses and mobile genetic elements in plants [4]. MicroRNAs (miRNAs) are single-stranded, noncoding sRNAs produced from endogenous transcripts with an imperfect self-complementary stem–loop structure [5, 6]. miRNAs play crucial regulatory roles in organisms by targeting specific mRNAs for cleavage or translational repression [7–9]. It has been reported that miRNAs affect various biological processes, such as the development of leaves, roots, and flowers, and affect grain filling [7, 10–12]. Grain filling is a critical phase in crop grain development. In rice, osa-miR167 regulates the auxin-miR167-ARF8-OsGH3.2 pathway, which functions during grain filling [13]. High-throughput sequencing revealed that the differentially expressed miRNAs between superior and inferior wheat grain are likely related to cell division, carbohydrate metabolism and hormone biosynthesis [14]. Han et al. [15] demonstrated that miR164, miR160, and miR169 show different expression profiles during grain filling, suggesting that their functions are coordinated during the different stages of wheat seed development. Similarly, Meng et al. [12] identified 873 miRNAs from wheat grain and suggested that many of them potentially regulate grain filling. Using a genome-wide investigation of miRNAs in different wheat tissues, Sun et al. [16] characterized 64 miRNAs that are expressed in developing wheat grain and that might play important functions in regulating grain development.

Furthermore, miRNAs that respond to nitrogen (N) deficiency in higher plants have been identified in previous studies [17–19]. In maize, miR169, miR399, miR518 and miR408 respond to N in the roots and leaves, but miR160, miR167, miR168 and miR395 respond to N deficiency only in the roots [18]. Several miRNAs, including miR167, miR171, miR398, miR827, miR408, and miR857, have been identified as being responsive to low-N conditions [20], and the wheat miRNA TamiR444a, which responds to nitrogen deficiency, is involved in plant adaptation to nitrogen starvation stress [21]. Zuluaga et al. [22] identified 84 novel and 161 conserved miRNAs in durum wheat in response to N starvation and found that ttu-miR169c and ttu-novel-61 were strongly downregulated under N-starvation conditions. Some miRNAs, such as miR159a, miR159b and miR399, showed significantly different expression profiles under low N treatment compared with high N treatment [23]. However, little information is available concerning the regulatory role of miRNAs in wheat grain in response to N deficiency, especially at different developmental stages.

Nitrogen is extremely important for plant growth and for agricultural productivity of many crop species [24]. Together with other N metabolites, nitrate can also act as a signal that regulates global gene expression [25]. In China, applying N fertilizer is an important agronomic practices to increase crop yield and economic productivity [26]. In addition to its effect on yield, N fertilizer is one of the key agronomic means of increasing grain protein content and improving wheat grain quality [27, 28]. However, excessive applications of N fertilizer have resulted in environmental pollution [29] and soil acidification [30]. Many efforts have been made to improve N fertilizer-use efficiency to reduce the waste of resources and to solve eco-environmental problems. Understanding the molecular mechanism of plant responses to N fertilizer applications might enable the development of crop varieties with increased nutrient-use efficiency and high grain yield and quality. To gain further insight into the role of wheat miRNAs that respond to different N fertilizer levels wheat grain development, the wheat cultivar Zhengmai 119 (ZM119), which has a high protein content, was grown under high nitrogen (HN) and low nitrogen (LN) conditions, and miRNA expression patterns were evaluated during grain development. The findings in this study will provide new insights into the regulatory function of wheat miRNAs that not only are associated with grain yield and quality but also respond to N supply levels.

## Results

### Grain characteristics

As shown in Table 1, the wheat cultivar ‘ZM119’ grown under HN conditions had a high grain yield, thousand-kernel weight, total protein content and protein fraction content (albumin, globulin, gliadin, and glutenin), whereas wheat grain from plants grown under LN conditions had low values. Correspondingly, the dough (rheological) characteristics of the wheat flour from the HN treatment had higher values than did the dough from the LN treatment. These results confirm that, compared with no N treatment, N fertilizer applications can increase grain yield and improve wheat grain quality.

**Table 1:** Grain characteristics of the wheat cultivar ‘ZM119’ grown under HN and LN conditions

Items	HN	LN
Thousand kernel weight (g)	50.23 ± 1.10^a^	48.52 ± 1.60^a^
Grain yield (kg ha^− 1^)	7184.6 ± 378.0^a^	4870.2 ± 28.1^b^
Protein content (mg g^− 1^)	153.38 ± 1.94^a^	112.36 ± 0.52^b^
Albumin content (mg g^− 1^)	31.69 ± 0.25^a^	24.94 ± 0.41^b^
Globulin content (mg g^− 1^)	10.32 ± 0.86^a^	7.33 ± 0.20^b^
Gliadin content (mg g^− 1^)	34.44 ± 0.10^a^	18.07 ± 0.75^b^
Glutenin content (mg g^− 1^)	61.88 ± 4.60^a^	50.45 ± 4.89^b^
Water absorption (%)	63.55 ± 0.40^a^	60.35 ± 0.49^b^
Dough development time (min)	6.95 ± 0.35^a^	1.90 ± 0.14^b^
Dough stability (min)	9.00 ± 1.41^a^	4.70 ± 0.98^b^

Data represent the mean ± standard deviation. Within the same row, mean values followed by different lowercase letters indicate significant differences at *P* < 0.05 (least significant difference).

### Deep sequencing of sRNAs in developing wheat grain from plants grown under two N fertilizer levels

To investigate the role of miRNAs in the developing wheat grain under different N fertilizer application levels, we sampled wheat grain at 7, 17, and 27 days after anthesis (DAA) under LN and HN conditions (three biological replicates each) and constructed a total of 18 sRNA libraries (Additional file 1: Table S1). The mean data of the three biological replicates are listed in Table 2.

**Table 2:** Sequencing data of the small RNA libraries constructed from developing wheat grain produced under two nitrogen fertilizer levels

Type		LN-7		LN-17		LN-27		HN-7		HN-17		HN-27	
		No.	%	No.	%	No.	%	No.	%	No.	%	No.	%
Redundant	Raw reads	10,621,127	100	11,752,733	100	10,898,065	100	10,652,837	100	10,948,017	100	10,475,211	100
	3 AD and length filter	3,809,346	36.38	6,656,958	56.05	5,115,582	46.94	4,985,140	46.62	6,192,961	56.53	3,646,829	34.73
	Other filters	381,771	3.57	898,931	7.72	1,320,520	12.09	332,596	3.13	799,749	7.31	780,664	7.46
	Clean reads	6,430,010	60.05	4,196,843	36.23	4,461,963	40.96	5,335,101	50.25	3,955,306	36.16	6,047,718	57.81
Unique	Raw reads	1,698,982	100	1,624,844	100	1,849,117	100	1,561,169	100	1,604,716	100	2,014,214	100
	3 AD and length filter	510,620	30.66	715,762	44.70	860,978	46.58	506,277	32.44	656,487	40.75	580,730	28.94
	Other filters	16,242	0.95	17,121	1.05	19,361	1.05	14,521	0.93	17,403	1.09	21,412	1.06
	Clean reads	1,172,120	68.39	891,960	54.25	968,778	52.37	1,040,371	66.63	930,826	58.16	1,412,072	70.00

No., number of reads; %, percentage of reads. The data are the means of three biological replicates.

**Fig. 1:**
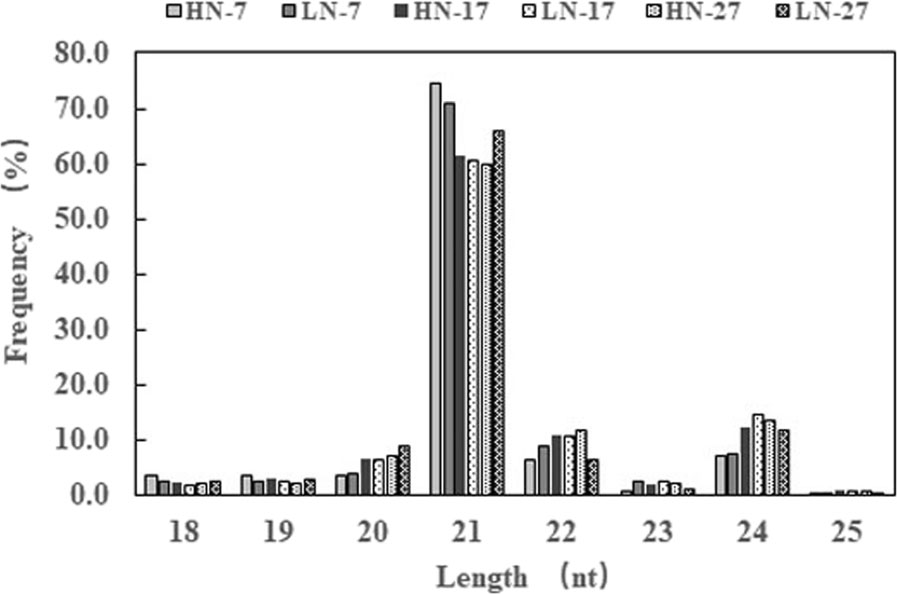
Length distribution of miRNAs in developing wheat grain of plants grown under two nitrogen application levels and sampled at 7, 17, and 27 DAA. HN-7, HN-17 and HN-27 represent grain at 7, 17, and 27 DAA under high nitrogen application levels, respectively. LN-7, LN-17 and LN-27 represent grain at 7, 17, and 27 DAA under low nitrogen application levels, respectively

After sequencing, 10,475,211 to 11,752,733 redundant raw reads were produced per library (Table 2). After removing the low-quality reads, 3,955,306 to 6,430,010 clean reads were obtained from each library, representing 36.16–60.05% of the total redundant raw reads. The number of unique raw reads ranged from 1,561,169 to 2,014,214, and the number of unique clean reads ranged from 891,960 to 1,412,072, representing 52.37–70.00% of the total unique raw reads. The majority of the miRNAs in the constructed libraries were between 21 and 24 nt in length (Fig. 1), with the 21-nt class (59.83–74.34%) being the most frequent of the total reads, followed by the 24-nt miRNAs (7.08–14.64%). The 21-nt miRNAs were more frequent in the HN-7 and LN-7 libraries, whereas the 24-nt miRNAs were well represented in the LN-17 and HN-27 libraries.

### Identification of known miRNAs and predicted novel miRNAs

**Fig. 2:**
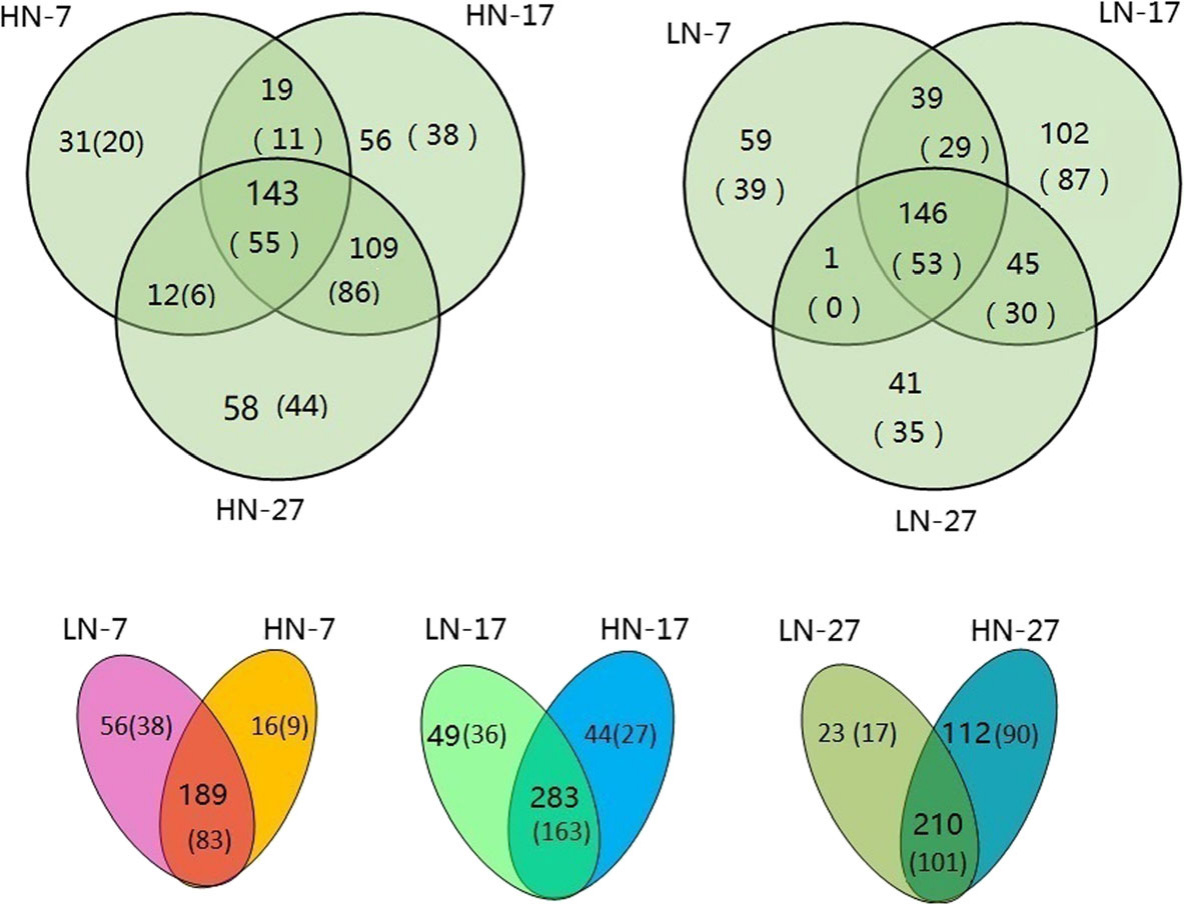
Venn diagram of differentially expressed miRNAs in different libraries at three grain developmental stages under high (HN) and low (LN) nitrogen treatment. HN-7, HN-17 and HN-27 represent grain at 7, 17, and 27 days after anthesis under high nitrogen application levels, respectively. LN-7, LN-17 and LN-27 represent grain at 7, 17, and 27 days after anthesis under low nitrogen application levels, respectively. The number outside (inside) the brackets represents the number of total (novel) differentially expressed miRNAs

To identify the conserved miRNAs in the constructed libraries in this study, the unique reads from each library were subjected to homology searches with known mature miRNAs from plants within miRBase (Additional file 2: Table S2). Additionally, novel miRNAs were predicted from unannotated sequences without similarity to known miRNAs (Additional file 2: Table S2, Additional file 3: Table S3). Since many miRNAs have not been deposited into miRBase, the novel miRNAs identified in this study were also compared with previously published wheat miRNAs [12, 14–16, 31–35]. In total, the sequences of 39 miRNAs from among the newly identified miRNAs were the same as those of previously found novel miRNAs (Table S2; the miRNAs are labeled in yellow). Most of the novel miRNAs had a relatively low expression level. The most abundant novel miRNAs were tae-miR9655-p5 and tae-miR2916-p3_2ss2TC17CA, which accounted for 24,656 reads and 27,735 reads in the LN-17 and LN-27 libraries, respectively. A total of 205, 327, and 322 unique miRNAs (of which 92, 190, and 191 were novel, respectively) were found in the HN-7, HN-17, and HN-27 libraries, respectively (Fig. 2). Additionally, a total of 143 miRNAs (55 novel ones) were found in the three HN-7, HN-17, and HN-27 libraries. The HN-7 and HN-17 libraries shared the same 19 miRNAs, of which 11 were novel. There were 245, 332, and 233 miRNAs (of which 121, 199, and 118 were novel, respectively) expressed in the LN-7, LN-17, and LN-27 libraries, respectively. We found that 146 miRNAs (including 53 novel ones) were expressed in all three LN libraries. When the effects of the two N fertilizer application levels were compared, LN-7 and HN-7 were found to share 189 expressed miRNAs, including 83 novel miRNAs. LN-17 and HN-17 shared 283 expressed miRNAs, of which 163 were novel. It is clear that the 17 DAA libraries (HN-17 and LN-17) contained more expressed miRNAs than did the other libraries.

### Differentially expressed miRNAs in the developing wheat grain

To identify the differentially expressed miRNAs in developing wheat grain, we analyzed the relative abundance of miRNAs in the HN-7, HN-17, and HN-27 libraries, and *P* ≤ 0.05 was used as a threshold to determine significant changes in expression during grain development. Similarly, the abundance of miRNAs in LN-7, LN-17, and LN-27 was also compared. A total of 179 miRNAs (91 known and 88 novel miRNAs) were identified in the comparisons of the three HN treatment libraries (Additional file 4: Tables S4–2 and S4–4). Additionally, 80 known and 76 novel miRNAs were identified among the three LN treatment libraries (Table S4–1, S4–3). All of these miRNAs belong to 51 miRNA families. As shown in Additional file 4 (Table S4–1), the known miRNAs that were differentially expressed between the three LN treatment libraries were divided into four groups. In group A-I, the expression of the identified miRNA showed an increasing trend with grain development; there were 17 miRNAs in this group, including two miR156s and three miR167s, of which tae-miR9674b-5p (Table S4–1) and tae-miR9622a-3p were the most abundant (Table S4–1). The tae-miR9666b-3p miRNA showed an increasing degree of expression in developing grain, with 3, 213, and 674 normalized reads in the LN-7, LN-17, and LN-27 libraries, respectively. This expression profile might indicate that these miRNAs have more important regulatory roles at later stages of grain development. Group A-II miRNAs show a decreasing expression profile with grain development, and there were 30 miRNAs in this group, including two miR159s, three miR171s, and four miR169s. In this group, ata-miR5168-3p was the most abundant, and ata-miR169d-3p_L-2R + 2 showed large fold-changes of − 3.81, − 1.18, and − 4.99 for the LN-17 vs, LN-7, LN-27 vs. LN-7, and LN-27 vs. LN-7 comparisons, respectively. The high levels of expression at the early stages of wheat grain development might be related to grain endosperm differentiation and cell division. The members of group A-III showed a single expression peak, with the highest abundance at 17 DAA. In this group, there were 18 miRNAs, of which tae-miR9655-3p was the most abundant. In addition, there were 15 miRNAs in groups A-IV, including three miR159s, four miR166s, and two miR319s (Table S4–1).

Similarly, there were four miRNA groups (for the known miRNAs) for the HN treatment libraries (Table S4–2). Group B-I contained 15 miRNAs, including two miR156s, one miR408, and three miR9666s. There were 30 miRNAs in group B-II belonging to 17 miRNA families. The 24 miRNAs in group B-III included three miR160s, three miR167s, and two miR531s, of which tae-miR160 (with an increase from 4129 reads to 6157 reads) was the most abundant. There were 22 miRNAs in groups B-IV, including four miR159s and four miR166s. The fold-changes for the HN-17 vs. HN-7 and HN-27 vs. HN-17 comparisons were − 2.23 and − 2.19, respectively, for tae-miR159a (Table S4–2). We noticed that some miRNAs exhibited the same expression profiles during grain development in both the HN and LN treatments. For example, tae-miR408_L-1, osa-miR827, tae-miR9666b-3p, tae-miR9666a-3p, and tae-miR9664–3p_L-1 were in group I (A-I, and B-I) in both the HN and LN treatments. Moreover, miR168, miR396, and miR530 were in group II (A-II, and B-II), and miR-528, miR-1127, and miR9655 were all in group III (A-III, and B-III) (Table S4–1, S4–2). These miRNAs exhibiting the same expression patterns between the two N application levels might be closely related to grain development. Of course, some miRNAs showed different expression profiles with respect to N application levels, which indicates that the regulatory role of these miRNAs might be related to N supply status. For example, ppt-miR894_L-1R + 1 was differentially expressed only in the HN treatment, whereas tae-miR9652-5p showed differential expression only in the LN treatment.

In addition, the expression patterns of novel miRNAs during grain development for the LN treatment and the HN treatment are shown in Table S4–3 and Table S4–4, respectively. Similar to the known miRNAs, the novel miRNAs identified among the LN treatments were also divided into four groups (Table S4–3). Group C-I contains 24 novel miRNAs, of which tae-miR2916-p5_2ss2AG17TG was the most abundant, and 15 miRNAs were not expressed in the LN-7 library. There were 18 and 31 novel miRNAs in groups C-II and C-III, respectively. Only three novel miRNAs were in group C-IV, of which tae-miR9674a-p5 was the most abundant (Table S4–3). Similarly, the differentially expressed novel miRNAs in the HN treatment libraries were also classified into four groups (Table S4–4). In group D-I, there were 30 novel miRNAs, of which 21 showed no expression in the HN-7 library but were highly expressed in the HN-27 library. There were 12 miRNAs in group D-II, of which tae-miR171a-p5 exhibited a large expression fold change (− 4.87) from 7 DAA to 17 DAA. There were 40 and 6 novel miRNAs found in groups D-III and D-IV, respectively (Table S4–4).

### Target gene functions of miRNAs differentially expressed during grain development

**Fig. 3:**
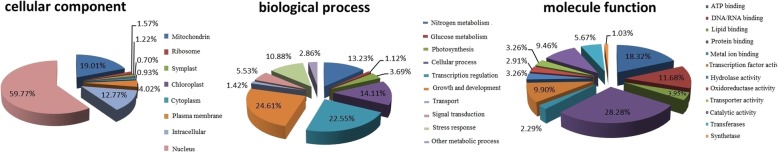
The major GO categories of “cellular component”, “biological process”, and “molecular function” for the predicted genes of all the differentially expressed miRNAs during grain development

Based on Gene Ontology (GO) assignments, we identified 3011 potential target genes for 354 differentially expressed miRNAs. The target functions of the miRNAs were analyzed according to the “biological process” (A), “cellular component” (B), and “molecular function” (C) GO categories. In the “biological process” category, the target genes were associated largely with 10 GO terms (Fig. 3), with the three most represented being “growth and development” (24.61%), “regulation of transcription” (22.55%), and “cellular process” (14.11%). Twelve molecular function GO terms were identified, with the three most frequent being “protein binding” (28.28%), “ATP binding” (18.32%), and “DNA/RNA binding” (11.68%). For “cellular component”, the most abundant GO term was “nucleus” (59.77%), followed by “mitochondrion”, with 19.01%.

To explore the roles of these differentially expressed miRNAs in grain development, we listed the known miRNAs and the novel miRNAs and their target functions in Tables 3 and 4, respectively (target gene IDs in Additional file 5: Table S5). It is clear that the target genes have diverse functions, and the encoded gene products include transcription factors, involve Skp-cullin-F-box (SCF) ubiquitin ligase activity, encode Cytokinin Response Factor 1 (CRF), encode protein kinases and encode heat-shock proteins (HSPs). tae-miR9674b-5p, tae-miR160, ata-miR166a-3p, and tae-miR159a are relatively abundant during all gain development stages (RAGS), and their target genes have multiple functions and encode proteins such as SCF proteins, protein kinase 20-like, serine carboxypeptidase, kinesin-2-like protein pollen semisterility 1 (PSS1) and MYB transcription factors (Table 3), indicating that these miRNAs might have multiple and important roles during all stages of grain development. The miRNAs osa-miR171a, ata-miR172c-3p, three miR396s (ata-miR396a-5p, ata-miR396e-5p, and ata-miR396c-5p), ata-miR393-5p_L-1R + 1 and ata-miR5168-3p were relatively more abundant during early stages (RAES) (HN-7 and LN-7) compared to later stages of development, and the gene products of their predicted targets include the DELLA proteins RGL2 (REPRESSOR OF GA1–3-LIKE 2), CRF1, proteins involved in auxin-activated signaling, serine-type endopeptidase, proteins that respond to gibberellin (GA) and leucine-rich-repeat (LRR) kinase. These miRNAs might play crucial regulatory roles in seed formation. Additionally, several miRNAs, including ata-miR528-5p, bdi-miR531_L-4R + 1_1ss5CT, tae-miR1127b-3p_1ss12TC, tae-miR9652-5p and tae-miR9655-3p, were relatively highly expressed during the middle stages of development (RAMS) (LN-17 and HN-17). The predicted targets of these miRNAs are LRR receptor kinases (involved in starch and sucrose metabolism), NAD(P)H dehydrogenase, UDP-glucuronate decarboxylase, proteins involved in transport, and L-fucosidase, which indicates that they might regulate starch accumulation and processes related to grain filling. The relative abundance of some miRNAs, including two miR156 family members (bdi-miR156h-3p_L + 1 and zma-miR156d-3p_L + 1_1ss9TC), tae-miR167b-5p, tae-miR319, osa-miR827, and tae-miR408_L-1, showed increased abundance at the later stage (RALS); their predicted targets were WRKY transcription factors, SCF proteins, MYB transcription factors, L-ascorbate oxidase, NB-ARC, and SYG1/PHO81/XPR1 (SPX), which suggests that these miRNAs might play important regulatory roles during later stages of grain development.

**Table 3:** Conserved miRNAs differentially expressed in developing wheat grain and their predicted target gene functions

Grain filling stages	miRNA	Abundance (RPM)			*P*- value	Abundance (RPM)			*P*- value	Target function
		LN-7	LN-17	LN-27		HN-7	HN-17	HN-27		
Relative abundance during grain filling stages (RAGS)	tae-miR9674b-5p	13,242	14,580	19,780	2.10E-05	14,337	14,831	20,374	3.32E-06	SCF ubiquitin ligase complex; calcium-dependent protein kinase 20-like
	tae-miR160	3696	6919	5125	3.34E-07	4129	6157	5376	5.90E-05	serine carboxypeptidase; transcription factor; Auxin reponse factor
	ata-miR166a-3p	9081	2818	4010	1.05E-08	8443	3171	4564	9.00E-09	kinesin-1-like protein PSS1; transport
	tae-miR159a	5372	1381	2706	1.28E-06	5191	1110	5056	2.89E-07	MYB; 40D repeat
Relative abundance at early stage(RAES)	osa-miR171a	1235	113	58	1.10E-09	1299	172	42	2.73E-07	DELLA proteins RGL2; malate dehydrogenase
	ata-miR172c-3p	162	104	0	1.65E-05	180	63	0	5.64E-06	cytokinin response factor1; DnaJ
	ata-miR396a-5p	1869	967	964	4.70E-07	1726	1085	762	1.40E-07	phosphatidylinositol 4-phosphate 5-kinase 1; glutamate dehydrogenase; response to GA
	ata-miR396c-5p	144	16	7	4.11E-06	134	22	3	2.34E-04	GTPase activity; HSP protein; RPM1
	ata-miR396e-5p	2264	772	709	7.40E-08	2366	931	722	2.87E-07	transcription factor TFIID; ribonuclease P activity
	ata-miR393-5p_L-1R + 1	325	45	37	6.88E-07	331	44	41	1.64E-07	auxin-activated signaling; protein TRANSPORT INHIBITOR RESPONSE 1; LRR kinase
	ata-miR167f-3p_L-1R + 1	61	26	19	5.86E-04	57	48	9	2.89E-04	ubiquitin ligase complex; lysosomal alpha-mannosidase
	ata-miR169e-5p	38	5	2	1.23E-06	63	5	1	8.42E-05	serine-type endopeptidase; peptidase
	osa-miR168a-5p	108	84	72	4.97E-03	110	78	73	5.45E-03	WRKY; Cul4-RING E3 ubiquitin ligase;
	osa-miR168a-3p_L-3	115	50	23	1.79E-04	162	47	39	5.70E-05	ubiquitin domain-containing protein DSK2b
	ata-miR169g-3p_L + 1R-1	250	7	0	4.17E-07	326	17	0	5.98E-06	ubiquitin protein ligase; RPM protein
	osa-miR171e-5p_2ss2GA21AG	344	12	0	1.51E-06	361	19	5	9.00E-10	protein binding
	osa-miR530-5p_L + 1	126	61	0	3.00E-04	134	109	30	8.85E-05	SCF ubiquitin ligase complex; fructose-bisphosphate aldolase
	ata-miR5168-3p	5692	638	454	2.00E-10	5409	559	511	0.00E+ 00	kinesin-1-like protein PSS1; Transcription factor;
	osa-miR530-5p_R + 1	52	36	0	3.00E-04	52	49	16	1.45E-03	fructose-bisphosphate aldolase; HSP70;
	osa-miR159a.1_L + 1	86	15	41	9.85E-03	94	9	85	8.26E-05	MYB; WS40 repeat protein
	tae-miR9776_L-1R + 1_1ss18AG	190	167	145	3.54E-03	215	158	143	6.85E-03	cell division; O-methyltransferase
	osa-miR166g-3p_L + 1_1ss21TC	118	14	16	1.10E-05	97	24	28	9.90E-06	transcription factor; transport
	tae-miR171a	696	125	126	1.00E-10	770	156	193	3.80E-06	DELLA protein RGL2-like; malate dehydrogenase
	ata-miR394-5p	286	42	98	3.46E-06	354	47	141	3.40E-09	SCF ubiquitin ligase complex; glutathione transferase activity
	ata-miR9772a-5p_L-2R + 2	47	21	16	1.17E-04	40	30	20	1.13E-02	SCF ubiquitin ligase complex
Relative abundance at middle stage(RAMS)	ata-miR528-5p	39	46	3	4.89E-04	28	38	23	1.99E-02	lectin-rich repeat receptor kinase
	bdi-miR531_L-4R + 1_1ss5CT	17	33	18	7.10E-03	12	38	23	1.27E-02	NAD(P)H dehydrogenase; ABC transporter
	ata-miR172b-3p	22	66	34	1.65E-05	26	36	20	1.35E-02	Cytokinin response factor; chaperone binding;
	tae-miR1127b-3p_1ss12TC	7	18	4	3.57E-02	2	26	13	2.84E-04	UDP-glucuronate decarboxylase; SCF ubiquitin ligase complex
	tae-miR9652-5p	3	28	0	3.46E-02	0	18	10	2.63E-02	retrograde transport
	tae-miR167c-5p	681	793	595	4.30E-02	638	790	336	2.95E-05	SCF ubiquitin ligase complex
	tae-miR9657a-3p	37	124	91	6.02E-05	23	87	68	5.70E-05	serine-type endopeptidase inhibitor activity; amino acid transport; WRKY
	tae-miR9655-3p	21	1031	312	4.10E-07	0	1099	333	2.78E-07	L-fucosidase; SCF ubiquitin ligase complex
	tae-miR9658-3p	24	37	20	1.10E-02	19	53	15	4.54E-04	SCF ubiquitin ligase complex; RPM1
Relative abundance at later stage (RALS)	bdi-miR156h-3p_L + 1	32	110	207	5.24E-04	23	103	219	9.19E-06	WRKY transcription factor
	zma-miR156d-3p_L + 1_1ss9TC	5	50	114	5.71E-05	9	28	64	3.78E-06	catalytic activity
	ata-miR167b-5p	111	576	889	1.38E-06	124	634	648	4.41E-07	SCF ubiquitin ligase complex
	tae-miR319	179	227	682	3.93E-06	130	199	1018	1.94E-06	MYB transcription factor; Dnaj protein
	osa-miR827	34	174	1002	7.10E-09	41	566	682	2.09E-07	SPX-domain-containing genes
	tae-miR408_L-1	28	207	213	6.71E-06	25	127	204	6.88E-05	L-ascorbate oxidase; LPEAT1-like
	tae-miR9666a-3p	0	439	4812	0.00E+ 00	0	492	911	3.68E-07	Alternative splicing regulator; regulatory protein NPR5
	tae-miR9666b-3p	3	213	674	4.59E-07	0	255	318	1.46E-07	DNA-directed RNA polymerase II subunit 1
	tae-miR9664–3p_L-1	15	29	55	4.78E-03	0	62	151	1.67E-07	RPM1 protein; zinc finger CCCH domain-containing; glucosidase
	hvu-miR399	0	24	66	2.40E-05	0	54	62	2.18E-05	NB-ARC domain; glucosyltransferase
	lus-miR159b_R + 1_1ss21CT	101	42	150	9.28E-04	78	22	179	2.10E-04	MYB
	osa-miR319a-3p.2-3p_R + 1	387	210	608	1.23E-05	507	193	537	3.02E-05	SCF ubiquitin ligase complex; MYB

^a^Abundance is indicated by normalized reads (reads per million of total miRNA reads (RPM)

^b^RPM ≥ 20 in these libraries

^c^The values are the means of three biological replicates

^d^*P*-value < 0.05 (5.00E-02) means a significant difference among different developmental stages

**Table 4:** Differentially expressed novel miRNAs associated with grain development and their target gene functions

Grian filling stages	miRNA	Abundance (RPM)			*P*- value	Abundance (RPM)			*P*- value	Target function
		LN-7	LN-17	LN-27		HN-7	HN-17	HN-27		
Relative abundance during grain filling stages (RAGS)	tae-miR2916-p5_2ss2AG17TG	1070	3667	9557	2.49E-06	1610	2067	2868	1.52E-05	protein serine/threonine kinase; glutathione S-transferase (GSTF)1; DELLA
	tae-miR2916-p3_2ss2TC17CA	2518	7110	27,736	5.92E-08	3507	4610	7049	1.66E-06	Peroxidase; fructose-bisphosphate aldolase; GTPase
	tae-miR9655-p5	2936	24,657	11,329	2.48E-05	668	20,120	11,554	1.30E-09	transcription factor; oxidation-reduction
Relative abundance at early stages(RAES)	tae-miR166d-p3	107	46	70	5.58E-03	83	56	80	1.97E-02	cell differentiation; kinesin-1-like protein PSS1
	PC-5p-30718_164	39	7	0	6.33E-05	49	2	15	4.94E-05	BRASSINOSTEROID INSENSITIVE 1; 1,3-beta-D-glucan synthase
	tae-miR815c-p5_2ss6CT21CT	107	92	21	3.62E-04	121	61	51	4.76E-04	UDP-arabinose 4-epimerase 1-like; transcription factor TGA2-like; Dnaj
	tae-miR9662b-p5	128	34	15	5.04E-07	153	31	18	4.56E-05	beta-fructofuranosidase
	tae-miR9662a-p5	95	62	46	5.53E-04	112	77	67	3.96E-02	beta-fructofuranosidase;
	tae-miR9672b-p5	125	33	0	1.57E-04	94	30	10	1.65E-03	LRR receptor-like serine/threonine-protein kinase FLS2-like
	tae-miR9779-p3	244	40	11	1.15E-03	361	43	93	9.91E-04	hexose transmembrane transport
Relative abundance at middle stages (RAMS)	tae-miR5384-p5	577	627	132	7.30E-06	579	686	489	1.07E-03	UDP-glycosyltransferase 83A1-like; ethylene-responsive transcription factor 1B
	PC-5p-3645_2157	33	175	148	1.88E-05	41	154	105	1.81E-05	Metalloendopeptidase; SCF ubiquitin ligase
	PC-3p-4780_1750	4	231	101	3.90E-05	0	135	104	1.09E-06	aspartic endopeptidase; transcription factor
	PC-5p-24289_247	2	23	3	1.20E-03	0	26	16	6.58E-04	NEDD8-specific protease; protein ECERIFERUM 1-like
	PC-5p-18073_397	0	66	26	3.34E-03	0	69	26	3.51E-06	beta-glucosidase; BRASSINOSTEROID INSENSITIVE 1-like
	PC-3p-21502_303	23	34	0	1.30E-03	38	30	25	1.03E-02	Metalloendopeptidase; aspartic-type endopeptidase;MYB
	tae-miR164-p3	68	159	143	1.24E-03	57	165	131	3.76E-03	auxin response factor 3-like; transcription factor IIB
	tae-miR164b-p3	16	62	18	1.71E-03	16	40	21	3.79E-03	auxin response factor 2-like; high-affinity nitrate transporter 2.1-like
	tae-miR1137a-p5_2ss9GA20GC	0	28	2	3.31E-02	0	13	2	1.77E-03	DNA mismatch repair protein MSH6
	tae-miR2275-p5	0	26	13	1.82E-04	0	28	2	1.66E-04	LRR receptor-like serine/threonine-protein kinase FLS2; BRASSINOSTEROID INSENSITIVE 1
	PC-5p-21100_312	0	35	17	2.14E-03	0	27	9	2.75E-03	glycerol-3-phosphate dehydrogenase; protein gar2
	PC-3p-15070_514	0	61	25	7.94E-04	0	44	41	2.64E-06	transcription factor
	tae-miR9653b-p3	6	36	5	8.36E-03	20	21	0	5.81E-04	protein binding
	PC-3p-11233_746	38	65	9	6.74E-05	22	58	33	1.38E-02	Metalloendopeptidase
	PC-3p-16735_445	10	43	13	3.99E-06	0	38	23	1.72E-05	O-acyltransferase WSD1-like
	PC-5p-12716_642	0	101	75	1.14E-04	0	111	79	6.00E-04	MAPK cascade; BRASSINOSTEROID INSENSITIVE 1
	tae-miR6300-p5	23	312	268	4.86E-05	25	258	253	1.42E-05	protein RPM1; response to nitrate
Relative abundance at later stages (RALS)	tae-miR2592bj-p3_2ss12TC19AT	82	193	380	6.17E-04	114	217	135	4.57E-04	protein serine/threonine phosphatase; transporter; protein RPM1-like
	PC-5p-31845_154	0	8	21	4.35E-03	0	8	11	5.36E-03	receptor-like serine/threonine-protein kinase SD1–6
	PC-5p-1231_4743	52	260	372	1.48E-06	40	339	453	1.35E-07	serine-type endopeptidase
	PC-5p-13909_573	0	47	66	6.93E-05	0	42	56	7.87E-05	GTPase
	PC-3p-13925_573	17	11	55	1.16E-06	5	9	87	6.72E-06	serine-type carboxypeptidase; valine-tRNA ligase
	tae-miR408-p5_1ss20GA	–	16	17	5.81E-04	–	16	22	9.12E-03	BRASSINOSTEROID INSENSITIVE 1

^a^Abundance is represented by normalized reads (reads per million of total miRNA reads (RPM))

^b^RPM ≥20 in these libraries

^c^The values are the means of three biological replicates

^d^*P*-value < 0.05 (5.00E-02) means a significant difference among different developmental stages

For the novel miRNAs (Table 4), two miR2916 members, tae-miR2916-p5_2ss2AG17TG and tae-miR2916-p3_2ss2TC17CA, not only presented the highest abundance in the grain but also increased in abundance during grain filling. These two miRNAs are predicted to target genes encoding serine/threonine kinases, glutathione S-transferase (GSTF) 1, peroxidase, fructose-bisphosphate aldolase, DELLA proteins, and proteins with GTPase activity, indicating that they may have multiple functions during grain development and could play important roles in regulating grain weight and nutrient accumulation. Many miRNAs, including tae-miR5384-p5, PC-5p-3645_2157, PC-3p-4780_1750, PC-5p-24289_247, PC-5p-18073_397, and PC-3p-21502_303, exhibited relatively high expression levels in the middle stage of wheat grain development and were predicted to target genes encoding UDP-glycosyltransferase, metalloendopeptidase, neural precursor cell-expressed developmentally downregulated 8 (NEDD8)-specific protease, and glucosidase.

**Fig. 4:**
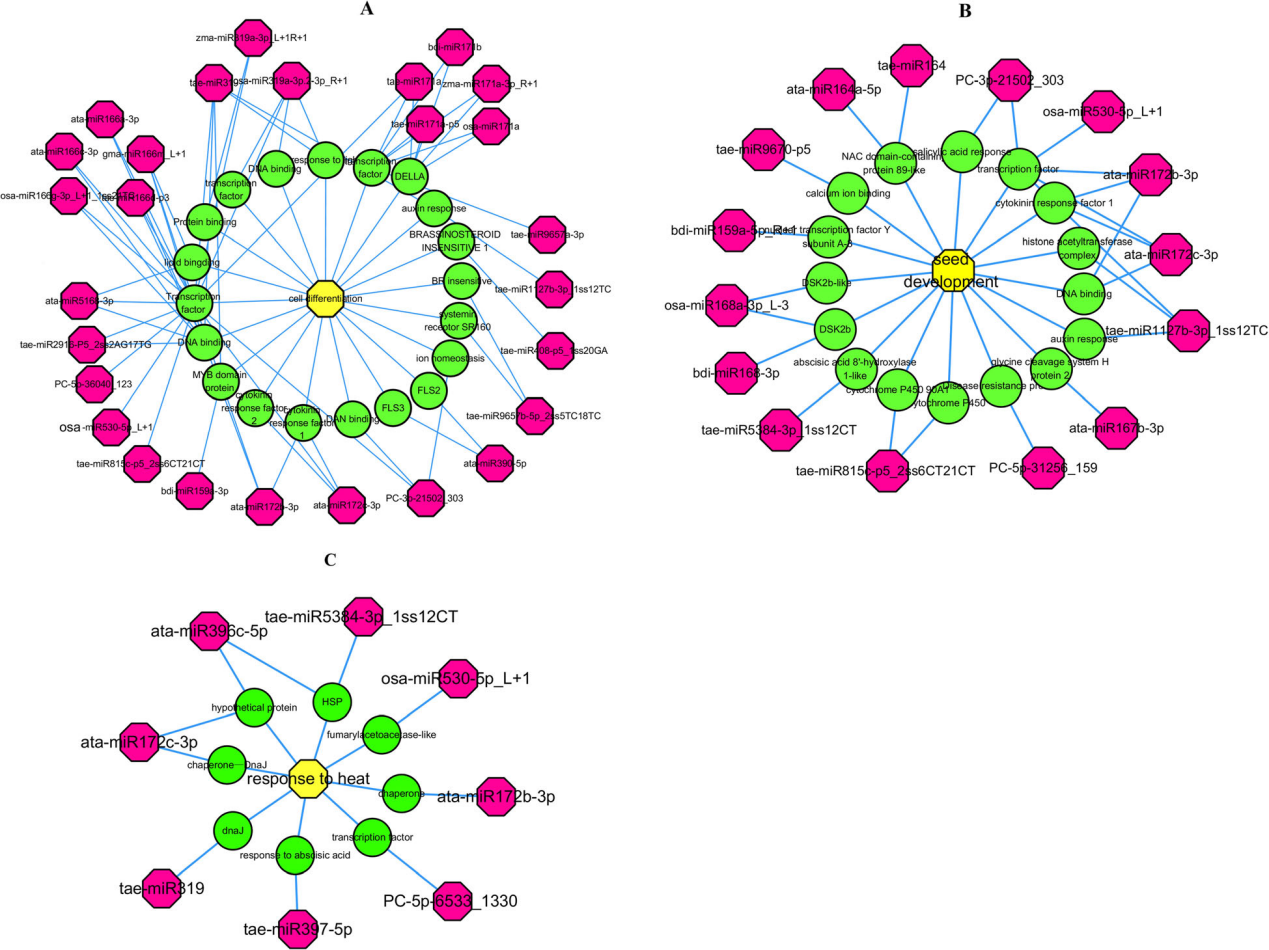
Relationships within a miRNA-gene-GO network for cell differentiation, seed development and response to heat stress as determined by Cytoscape. The red, green, and yellow colors indicate miRNAs, target gene functions and GO terms, respectively

Cell differentiation plays an important role in determining wheat grain size. To clarify the potential regulatory mechanisms underlying cell differentiation, we constructed a miRNA target gene GO network by selecting miRNAs and their target genes based on their GO annotation (Fig. 4a).

There are 27 miRNAs and 20 genes in this network, with five, five, and three miRNAs belonging to the miR171, miR166, and miR319 families, respectively. It is clear that miR166 members target mainly transcription factor-encoding and nucleic acid-related genes, and the miR171 family members target genes mainly involved in the hormone response. Additionally, we constructed a network with miRNAs that had GO annotations related to seed development (Fig. 4b). Fifteen miRNAs and 16 genes are included in this network, including ata-miR167b-3p, which is predicted to target glycine cleavage system H protein 2. The tae-miR815c-p5_2ss6CT21CT miRNA might regulate grain development by targeting cytochrome P450s, which play roles in carbohydrate metabolism. Additionally, a network including eight miRNAs and eight target genes was constructed with GO annotations related to the heat stress response (Fig. 4c). This network shows that the miRNA targets include genes that encode chaperones, transcription factors, HSPs, DnaJ, and components of the abscisic acid (ABA) pathway, indicating that the miRNAs might participate in the heat stress response by regulating various pathways. Additionally, miR172 members might play an important role in the response to heat stress by regulating chaperones.

### miRNAs that are differentially expressed between the two N levels and their potential target gene functions

A comparison between HN and LN revealed 40 (25 known and 15 novel), 44 (26 known and 18 novel), and 100 (48 known and 52 novel) differentially expressed miRNAs at 7 DAA, 17 DAA, and 27 DAA, respectively (Additional file 6: Table S6–1, S6–2, and S6–3). To further explore the role of these miRNAs in grain development and the response to N supply levels, the criteria of log_2_(fold change) ≥1.0 and reads per million of total miRNA reads (RPM) ≥20 were further used to select the differentially expressed miRNAs in the constructed libraries. For the LN-7 vs. HN-7, LN-17 vs. HN-17, and LN-27 vs. HN-27 comparisons, the ratios of upregulated miRNAs were 2/6, 0/6, and 5/10 for the known miRNAs, respectively, and were 5/5, 2/3, and 8/24 for the novel miRNAs, respectively (Table 5).

**Table 5:** Number of miRNAs that show differential expression between the HN and LN treatments

Library	Known			Novel			Total		
	Up	Down	Total	Up	Down	Total	Up	Down	Total
LN-7 vs. HN-7	2	4	6	5	0	5	7	4	11
LN-17 vs. HN-17	0	6	6	2	1	3	2	7	9
LN-27 vs. HN-27	5	10	15	8	16	24	13	26	39

The differentially expressed miRNAs and their target gene functions are listed in Table 6. For the known miRNAs, the abundance of sbi-miR399a was significantly higher in the HN treatments (HN-7 and HN-17) than in the LN treatments (LN-7 and LN-17), and tae-miR9664–3p_L-1 showed significantly higher expression in the HN treatment than in the LN treatment at 17 and 27 DAA. Additionally, ata-miR172b-5p had a higher expression level in the LN-27 treatment than in the HN-27 treatment. For the novel miRNAs, we found more differentially expressed miRNAs at the late developmental stages (LN-27 and HN-27). PC-5p-26413_215 had significantly higher expression in the LN treatment than in the HN treatment at the three grain developmental stages and potentially regulates BRASSINOSTEROID INSENSITIVE 1-associated receptor kinase 1 (BAK1) and LRR receptor-like serine/threonine-protein kinase. We found that 16 novel miRNAs were significantly upregulated in the HN-27 library compared to the LN-27 library, and these include two tae-miR2916 family members (tae-miR2916-p3_2ss2TC17CA and tae-miR2916-p5_2ss2AG17TG) that show fold-changes of 1.74–1.98 between LN-27 and HN-27. Both PC-3p-15988_474 and PC-5p-6533_1330 were more abundant in LN-27 than in HN-27 and potentially function by targeting defense (peroxidase activity)- and transcription factor-encoding genes. PC-5p-36040_123 also showed higher expression in LN-27 compared to HN-27, and its predicted target gene product is disulfide isomerase, which indicates that it might be related to the regulation of grain storage and protein quality.

**Table 6:** Differentially expressed miRNAs between HN and LN treatments and their predicted target gene functions

Cluster	miRNA ID	Abundance (RPM)		*P*- value	Abundance (RPM)		*P*- value	Abundance (RPM)		*P*- value	Target function
		LN7	HN7		LN17	HN17		LN27	HN27		
Known	bdi-miR156b-5p	19	81	4.27E-02	–	–		–	–		–
	tae-miR159a-p5	18	38	1.75E-02	–	–		–	–		–
	osa-miR159a.1_L + 1	–	–	–	–	–		41	85	1.39E-02	MYB; WD40 repeat-like protein
	ata-miR164a-5p	–	–	–	–	–		19	54	4.90E-02	glutathione S-transferase; MAP kinase;
	ata-miR169d-5p	–	–	–	–	–		7	23	2.13E-02	protein serine/threonine kinase; ABC transporter C family member 13
	ata-miR169d-3p_L-2R + 2	–	–	–	–	–		27	92	8.22E-05	galactinol--sucrose galactosyltransferase 2; E3 ubiquitin-protein ligase
	ata-miR172b-5p	–	–	–	–	–		43	2	1.38E-03	–
	sbi-miR399a	27	113	5.40E-03	9	109	1.05E-03	–	–		Oxidoreductase; transcription factor
	hvu-miR399	–	–	–	24	54	4.73E-03	–	–		UDP-glucosyltransferase; transcription factor
	ata-miR528-5p			–				3	23	2.20E-02	lectin-rich repeat receptor kinase
	osa-miR530-5p_R + 1_1ss20AG	5	31	3.22E-02	0	11	2.57E-03	–	–		–
	osa-miR530-5p_L + 1	–	–	–	–	–		0	30	4.62E-03	fructose-bisphosphate aldolase; SCF ubiquitin ligase complex
	osa-miR827	–	–	–	174	566	2.36E-05	–	–		spx-domain-contain; Phenylalanyl-tRNA synthetase
	tae-miR9653a-3p	–	–	–	–	–		181	0	9.46E-04	–
	tae-miR9659-3p	55	0	6.03E-03	_	_		_	_		–
	tae-miR9661-5p_1ss14GA	–	–	–	_	_		22	0	5.49E-03	–
	tae-miR9664–3p_L-1	15	0	2.00E-02	29	62	4.34E-03	55	151	2.29E-03	RPM1; Gibberellin receptor; peptidyl-prolyl cis-trans isomerase;
	tae-miR9666a-3p	–	–	–	–	–		4812	911	1.38E-07	Alternative splicing regulator; regulatory protein NPR5-like
	tae-miR9666b-3p	–	–	–	–	–		674	318	2.57E-04	stress response
	tae-miR9672a-3p_L + 2R-2	–	–	–	15	41	4.01E-02	19	5	5.50E-02	replication factor C subunit 1
	tae-miR9774_L + 2	–	–	–	–	–		107	42	1.12E-03	–
	tae-miR9777_L + 2	–	–	–	–	–		14	46	2.94E-02	geraniol 8-hydroxylase-like; SCF ubiquitin ligase
Novel	PC-3p-40666_99	32	0	1.06E-02	–	–		–	–		transcription factor activity
	PC-5p-11080_759	72	21	1.73E-02	–	–		0	30	3.60E-03	–
	PC-5p-26413_215	23	4	3.03E-02	23	9	4.43E-02	11	0	5.99E-03	BRI 1-associated receptor kinase 1-like; LRR receptor-like serine/threonine-protein kinase
	PC-3p-43479_87	24	5	4.85E-02	–	–		–	–		–
	tae-miR9655-p5	2936	668	2.87E-04	–	–		–	–		oxidation-reduction process; transcription factor
	PC-5p-16963_436	–			32	91	8.60E-04	0	74	1.78E-02	–
	tae-miR1137a-p3_1ss9CT	–			9	25	1.92E-02	–	–		–
	PC-3p-15988_474	–			–	–		206	51	9.29E-04	peroxidase activity
	PC-5p-6533_1330	–			–	–		269	47	3.16E-03	regulation of transcription
	PC-3p-33976_137	–			–	–		25	0	3.29E-03	RPM1
	PC-5p-21478_303	–			–	–		77	21	2.41E-02	lectin S-receptor-like kinase
	PC-5p-36040_123	–			–	–		24	9	3.25E-02	protein disulfide isomerase; endopeptidase
	tae-miR9666a-p5	–			–	–		101	30	3.22E-05	–
	tae-miR2916-p3_2ss2TC17CA	–			–	–		27,736	7049	1.51E-03	fructose-bisphosphate aldolase; PRM1; peroxidase
	tae-miR2916-p5_2ss2AG17TG	–			–	–		9557	2868	5.04E-03	glutathione S-transferase; DELLAL
	tae-miR2592bj-p3_2ss12TC19AT	–			–	–		380	135	1.72E-02	protein serine/threonine kinase activity; RPM1;
	PC-3p-21085_312	–			–	–		3	44	1.36E-03	–
	PC-3p-21502_303	–			–	–		0	25	1.97E-03	metalloendopeptidase activity; MYB
	PC-5p-9458_913	–			–	–		0	133	3.25E-03	–
	PC-5p-4529_1828	–			–	–		62	144	1.15E-02	–
	PC-3p-11233_746	–			–	–		9	33	2.87E-02	metalloendopeptidase activitys
	tae-miR9783-p3_2ss5AG18AC	–			–	–		4	59	4.41E-04	protein RPM1-like
	tae-miR9657c-p5_1ss20TC	–			–	–		17	114	5.39E-04	Leucine-rich receptor-like protein kinase;
	tae-miR5384-p5	–			–	–		132	489	5.66E-04	UDP-glycosyltransferase; transcription factor
	tae-miR9779-p3	–			–	–		11	93	2.83E-03	hexose transmembrane transport
	tae-miR815c-p5_2ss6CT21CT	–			–	–		21	51	4.56E-03	UDP-arabinose 4-epimerase; TGA2-like; Dnaj
	tae-miR1127b-p5_1ss3TC	–			–	–		0	20	6.05E-03	–
	tae-miR1122c-p3	–			–	–		17	46	4.34E-02	phosphatase HAL2; protein kinase

^a^Abundance is represented by normalized reads (reads per million of total miRNA reads (RPM))

^b^RPM ≥20 in these libraries

^c^The values are the means of three biological replicates

^d^*P*-value < 0.05 (5.00E-02) means a significant difference between HN and LN

**Fig. 5:**
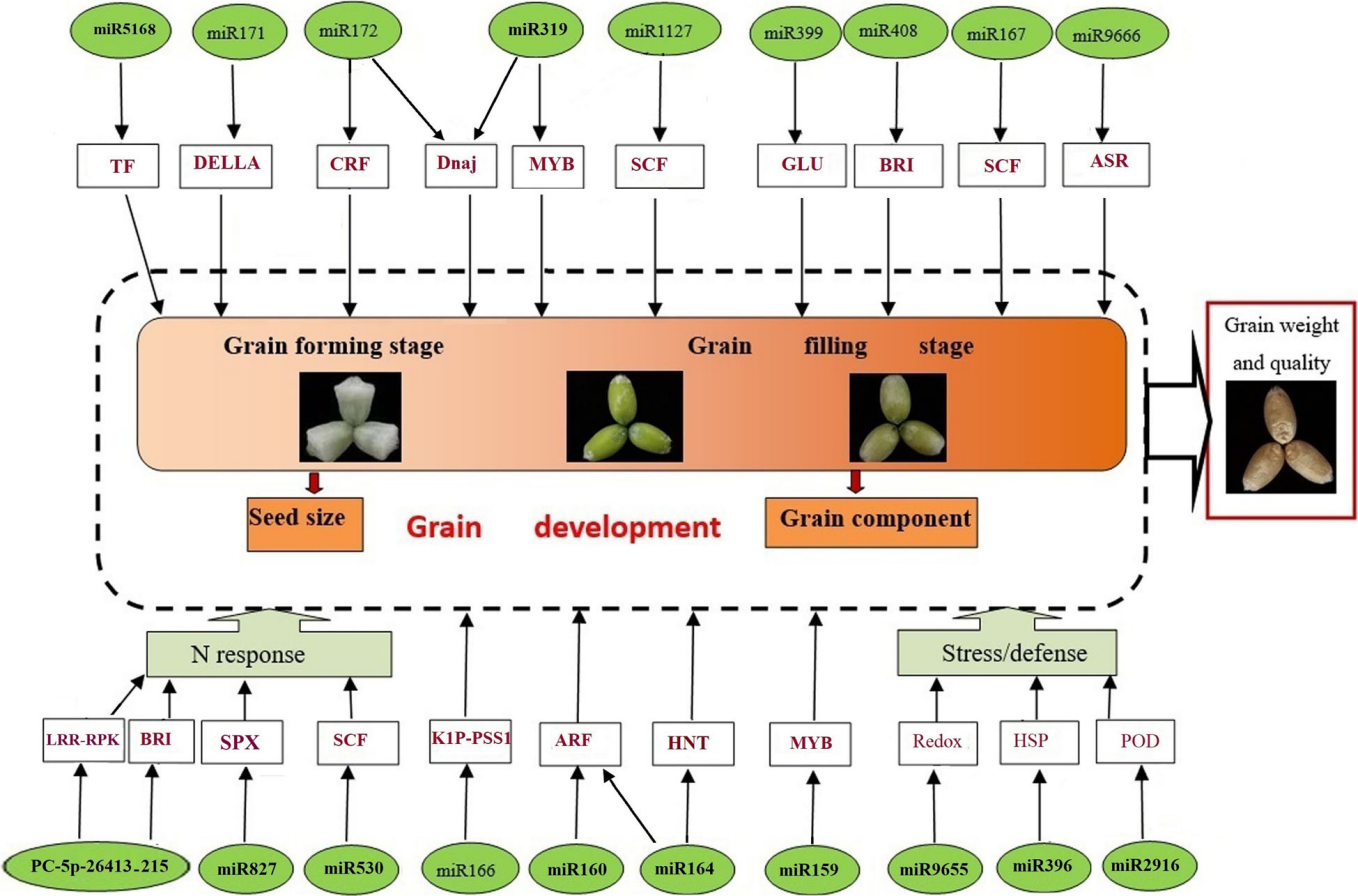
Model for miRNAs and their target genes associated with grain development and the response to N levels. The black letters in green circles indicate miRNAs, and the red letters in black boxes indicate the target gene function. The black arrows represent the direction of regulation. TF, transcription factor; CRF, cytokinin response factor; SCF, Skp-cullin-F-box; GLU, glucosyltransferase; BRI, brassinosteroid insensitive; ASR, alternative splicing regulator; LRR-RPK, leucine-rich-repeat receptor-like protein kinase; K1P-PSS1, kinesin-1-like protein PSS1; ARF, auxin response factor; HNT, high-affinity nitrate transport; Redox, reduction-oxidation; HSP, heat-shock protein; POD, peroxidase

A network of the miRNAs that are differentially expressed between the HN and LN treatments was constructed on the Cytoscape platform v.3.6.0 (https:cytoscape.org) [36] (Additional file 7: Figure S1). The differentially expressed miRNAs have multiple potential functions, such as targeting carbohydrate metabolism, nitrogen metabolism, signal transduction, hormone responses, and defense. However, it is clear that eight miRNAs, tae-miR2916-p5_2ss2AG17TG, PC-3p-4066_99, tae-miR5386-p5, PC-5p-26413_215, ata-miR164a-5p, tae-miR9655-p5, PC-3p-21502_303, and sbi-miR399a, have the same target—genes encoding transcription factors. Apart from carbohydrate metabolism and signal transduction, five miRNAs, tae-miR2916-p3_2ss2TC17CA, PC-3p-15988_474, osa-miR530-5p_L + 1, tae-miR2592bj-p3_2ss12TC19AT, and ata-miR528-5p, were predicted to target genes that function in transport. We also noticed that tae-miR9664–3p_L-1 not only has multiple potential functions but also is linked to the transcription factor group and the transport group via the RPP13 and RPM1 proteins. The results of these analyses indicate that the differentially expressed miRNAs might respond to different nitrogen fertilizer conditions by regulating multiple metabolic pathways. Additionally, transcription factors and transport proteins may play important roles in the response to N supply status. We also constructed a model to show how the regulation of differentially expressed miRNAs is related to grain development and the response to N supply (Fig. 5).

### Validation of identified miRNAs and their target genes

**Fig. 6:**
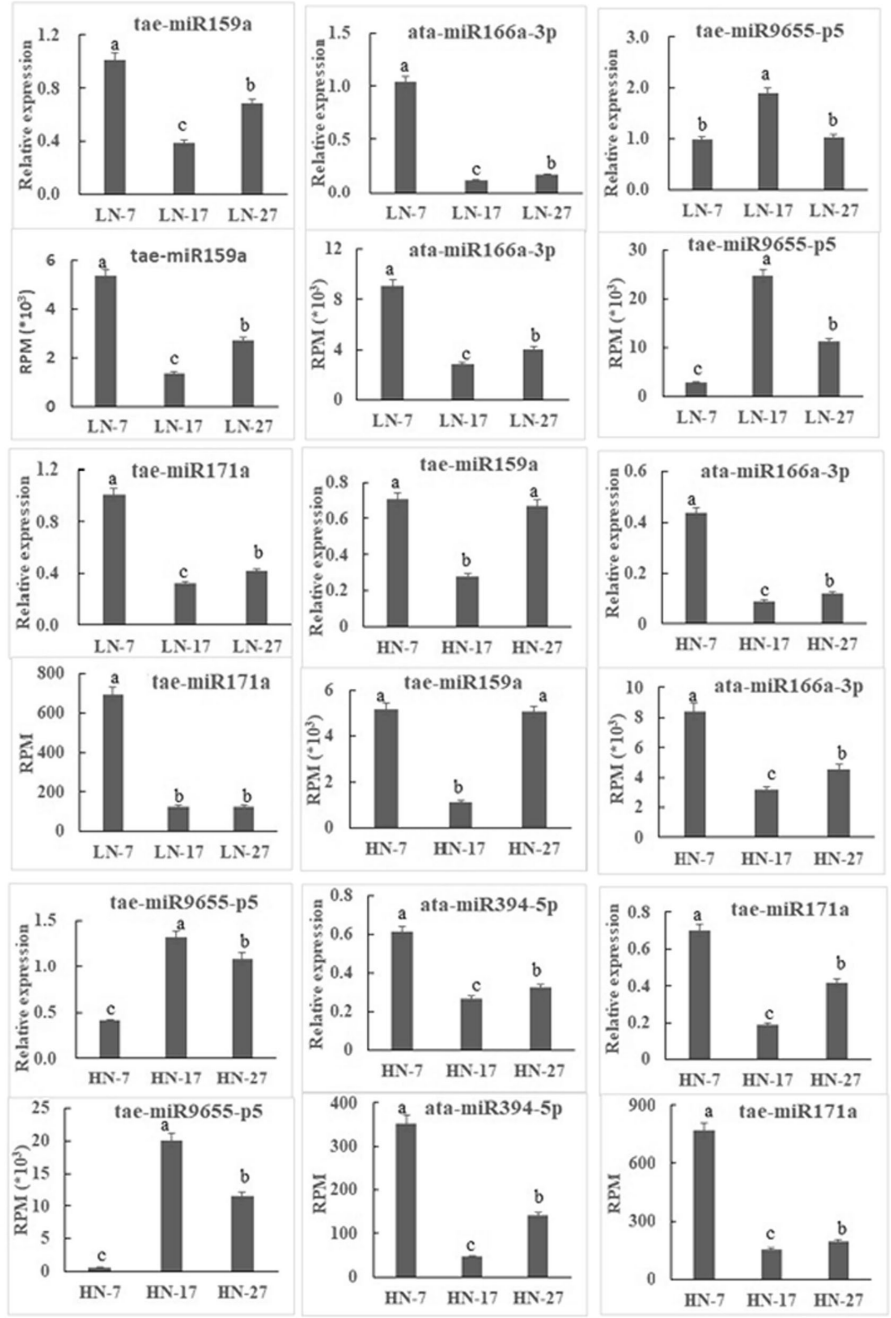
Verification of the expression patterns of nine miRNAs present in developing wheat grain. The different lowercase letters above the columns indicate significant differences (*P* < 0.05)

To confirm the miRNA expression in this study and to validate the deep sequencing results, we randomly selected four and five miRNAs (including one novel) from the LN and HN libraries, respectively, for quantitative real-time polymerase chain reaction (qRT-PCR) assays. The expression patterns of these miRNAs were similar to those obtained from deep sequencing, suggesting that the sRNA sequencing results obtained in this study are reliable (Fig. 6).

**Fig. 7:**
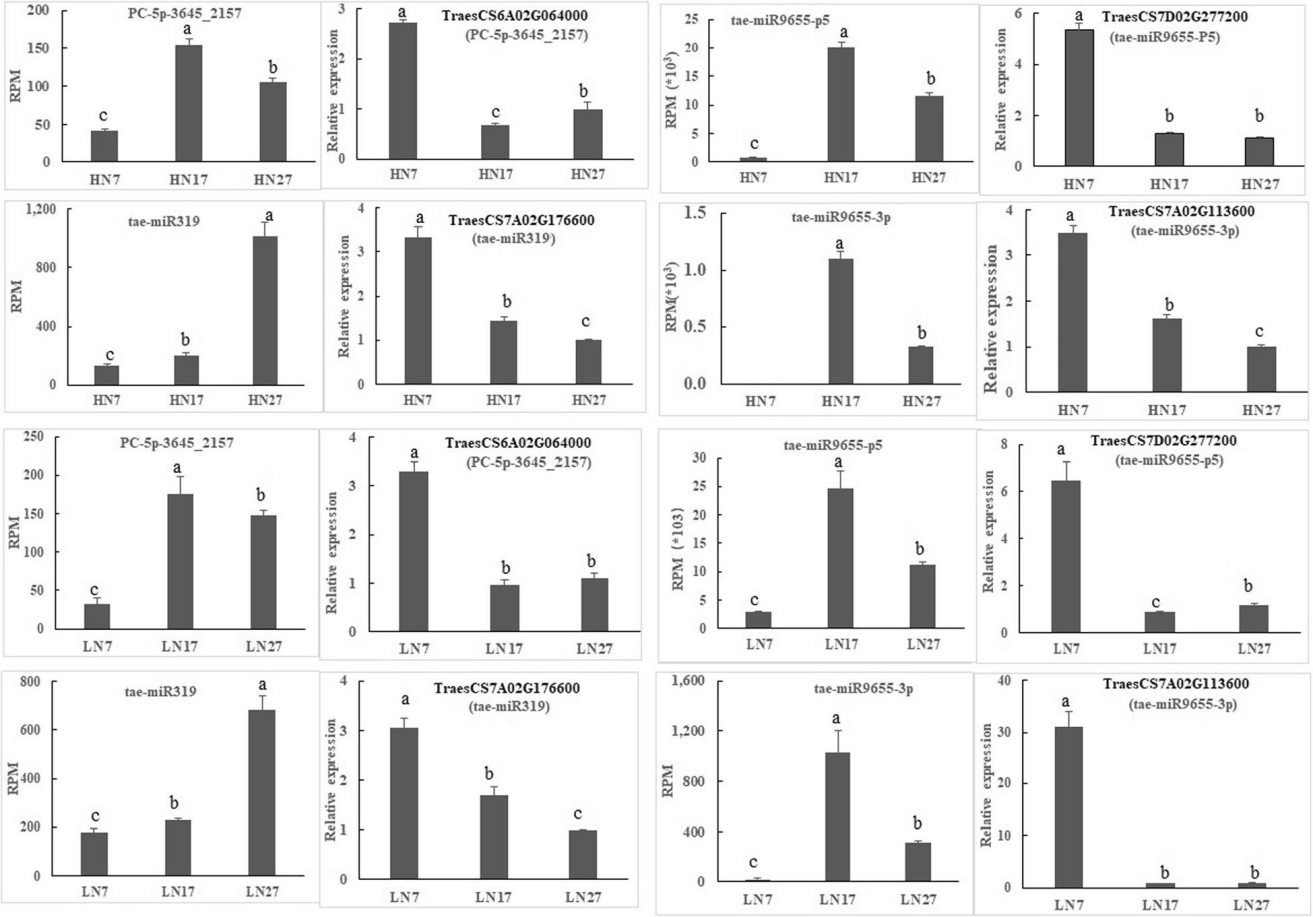
Verification of the expression patterns of miRNA target genes in developing wheat grain. The different lowercase letters above the columns indicate significant differences (*P* < 0.05)

To verify the expression patterns of the potential miRNA targets, four target genes (the gene IDs and the primer sequences are given in Additional file 8: Table S7) were selected for gene-specific qRT-PCR under the HN and LN treatments (Fig. 7). The expression levels of PC-5p-3645_2157, tae-miR9655-p5, tae-miR9655-3p, and tae-miR319 were found to be upregulated with grain development, and their predicted target genes showed downregulated expression patterns. These results indicate that a negative relationship exists between target gene expression and their corresponding miRNA expression profiles.

## Discussion

### Grain characteristics under different N treatments

Nitrogen plays an important role in plant growth and agricultural production [24, 26], and N fertilization is an effective agronomic practice to increase wheat grain yield and improve grain processing quality [27, 28]. Here, HN treatment resulted in higher grain yields, protein content and dough development time, which is consistent with the results of previous reports [27, 28, 37]. We also observed that the gluten (gliadin and glutenin) content (27.80 mg g^−1^) increased more than did the albumin and globulin content (9.74 mg g^−1^) under HN treatment, indicating different responses of the grain protein component to N fertilizer. According to the national standards of high-quality wheat [38], the grain under HN conditions had a high protein content, and its dough rheological characteristics (water absorption, dough development time and stability) met the standard for strong-gluten wheat, which confirms that N fertilizer applications can improve grain processing quality.

### miRNAs involved in wheat grain development

A better understanding of the regulatory roles of miRNAs in grain development and the response to nitrogen application levels will provide new perspectives for improving grain yield and quality. Previous studies have identified several conserved miRNAs in wheat grain, such as miR159, miR160, miR164, and miR156 [12, 15]. In this study, we also identified several highly conserved miRNA families, such as miR156, miR396, miR160, miR172, and miR164, indicating that these miRNAs are abundant in wheat grain and potentially play important regulatory roles in grain development. The reproductive stage of wheat from flowering to maturity includes the grain formation and filling stages; the number of endosperm cells in wheat is directly related to grain yield and quality [39]. A rapid increase in the number of endosperm cells occurs at 6–9 DAA, and the final number is determined by 14–18 DAA [39]. Members of the osa-miR171a, ata-miR172c-3p, miR396 (ata-miR396e-5p, ata-miR396a-5p, and ata-miR396c-5p) and ata-miR5168-3p families were the most abundant miRNAs at 7 DAA, suggesting that these miRNAs might be involved in the early stages of grain development. Higher abundances of miR171a and miR172 members were also found in the early developing grain by Meng et al. [12]. Additionally, by analyzing the miRNA target gene GO terms, we identified 27 miRNAs with target gene products related to cell differentiation (Fig. 4), including osa-miR171a and ata-miR172c-3p. It has been reported that miR172 members control floral organ identity in rice, maize, and barley by regulating the expression of APETALA2 (AP2)-like genes [40–42]. The floral and seed development of transgenic rice overexpressing miR172b were defective; the plants also presented relatively low spikelet fertility and reduced seed weight [42]. Here, the predicted target of ata-miR172c-3p is the CRF gene. CRFs compose a small subset of APETALA2/ethylene response factor (AP2/ERF) transcription factors that regulate plant development as part of the cytokinin signal transduction pathway [43], suggesting that ata-miR172c-3p might be involved in wheat seed development via cytokinin regulation. A positive correlation between cytokinin level during grain development and final grain weight has been reported in maize and wheat [44, 45]. Apart from cell differentiation, we also found that the target gene products of ata-miR172c-3p (DnaJ) and ata-miR172b-3p (chaperone binding) are involved in seed development and the response to heat stress (Fig. 4), which might indicate that they play important regulatory roles in determining wheat grain size and weight. Ma et al. [46] found that the miR171-SCL module plays a key role in mediating gibberellin (GA)-DELLA signaling in the coordinated regulation of leaf growth and chlorophyll biosynthesis in the light. We also predict that potential targets for osa-miR171a are genes that encode proteins such as the DELLA protein RG2-like, which is a regulator in the GA signaling pathway. It has been shown that miR396 members affect rice yield by regulating grain size [47], and the base substitution from TT to AA of the target genes can counteract the suppression by miR396, resulting in the activation of the hormone response and an increase in grain size [48, 49]. Schommer et al. [50] also found that the transcription factor TCP4, which is a direct regulator of miR396b, can repress cell proliferation by activating various pathways. Here, three miR396s (ata-miR396e-5p, ata-miR396a-5p, ata-miR396c-5p) were relatively abundant at 7 DAA, and the encoded proteins of the target genes function in response to GA, function as transcription factors, are GTPases, and act as protein kinases, suggesting that the regulation of these miRNAs might be related to grain size. All these results suggest that these miRNAs may play crucial roles in regulating grain endosperm cell development, which is directly related to grain size. However, additional research is needed to explore the target genes and their functions.

Grain filling is an important process in kernel development and ultimately determines kernel weight and flour quality. It has been suggested that 14–18 DAA is a major transition point for the formation of the wheat starchy endosperm and essentially marks the end of endosperm cell division and the deposition of starch and gluten protein [39]. Meng et al. [12] identified some miRNAs that might be involved in the control of grain filling; these miRNAs are involved in various biological processes by targeting many different wheat genes, such as those involved in carbohydrate metabolism, protein metabolism, transport, and transcription. In our study, we identified several conserved miRNAs and novel miRNAs that not only were highly abundant in wheat grain but also were significantly up- or downregulated during grain development; examples include tae-miR160 and ata-miR166a-3p. The gene products of the potential gene targets of these miRNAs include transcription factors, auxin response factors (ARFs), serine carboxypeptidases, kinesin-1-like protein PSS1, and proteins involved in transport. It is well known that miR160 directs the cleavage of several ARF mRNAs to regulate plant growth and seed germination [51, 52]. Expression of a miR160-resistant version of ARF17 in plants causes dramatic developmental defects, and overexpression of miR160 results in fewer starch granules and disorganized root tips [52, 53]. Analysis of the conservation and diversification of the miR166 family in plants showed that miR166 members play a wide and important regulatory role in seed development [54]. Therefore, these data suggest that these miRNAs have multiple regulatory functions in grain filling. In this study, we also identified some significantly upregulated novel miRNAs (such as ae-miR5384-p5, PC-5p-3645_2157, PC-3p-4780_1750, PC-5p-24289_247, PC-5p-18073_397, and PC-3p-21502_303) in the middle stage of grain development (17 DAA). The predicted proteins encoded by the targeted genes include UDP-glycosyltransferase, NEDD8-specific protease, metalloendopeptidase, and glucosidase, which indicate that these upregulated miRNAs might be related to carbohydrate and nitrogen metabolism, which would affect starch deposition and protein accumulation.

Some miRNAs, such as tae-miR319, hvu-miR399, and miR9666, showed little or no expression during the early stage (7 DAA) but then became upregulated as grain development progressed, and these miRNAs were most abundant at 27 DAA. The potential encoded proteins of the genes targeted by these miRNAs include MYBs, DnaJ, NB-ARC domain-containing proteins, and alternative splicing regulators. miR319 is a conserved microRNA that regulates TCP transcription factors involved in various developmental pathways, such as hormone biosynthesis, signaling, and leaf development and senescence [55, 56]. We also predicted that one of the potential targets of miR319 in the response to heat stress is the gene encoding the DnaJ protein. High-temperature stress at grain filling is a major problem in the production of high-quality wheat, and further exploration of the internal mechanism of these miRNAs in response to heat stress would be a potential way to reduce the effects of high-temperature stress.

Grain quality is a complex trait and includes not only protein content but also wheat gluten polymer structure [57]. Chen et al. [58] reported that cysteine and methionine metabolism pathways have an important role in storage protein biosynthesis via the synthesis of sulfur-containing amino acids. Here, KEGG (Kyoto Encyclopedia of Genes and Genomes) pathway analysis showed that the target genes of bdi-miR531_L-4R + 1_1ss5CT and ata-miR396e-5p were annotated as involved in cysteine and methionine metabolism. The formation of disulfide (SS) bonds regulated by protein disulfide isomerase is a key factor that determines gluten protein polymerization [57, 59]. We identified one novel miRNA, PC-5p-36040_123, which is predicted to target protein disulfide isomerase, that was significantly downregulated at HN-27. The downregulated expression under high nitrogen treatment is predicted to cause an increase in disulfide isomerase activity and to promote grain protein polymer formation. In addition to SS bonds, hydrogen or electrostatic bonds or other noncovalent bonds also participate in the formation of gluten protein polymers [57]. Additional information concerning the regulatory function of the identified miRNAs and their relationship to the protein polymer structures in grain and flour is needed.

### Differentially expressed grain miRNAs in response to nitrogen application levels

Many miRNAs have been identified that respond to nutrients and abiotic stress, such as salt and drought stress [60]. In this study, we identified 50 miRNAs (including 28 novel ones) that showed significant differential expression between HN and LN treatments. The expression of miR399 is upregulated by phosphorus (P) deficiency [61] and is related to P homeostasis by regulating the expression level of the target *PHO2* gene, which encodes a ubiquitin-conjugating E2 enzyme, UBC24 [62]. Baek et al. [63] also found that AtMYB2, a transcription factor, can regulate miR399 expression levels in response to P starvation. Similarly, Zhao et al. [23] reported that TamiR399 was downregulated under conditions of N deficiency. In our study, we found that the expression of two miR399s (sbi-miR399a and hvu-miR399) was significantly downregulated in the LN treatment. The two miRNAs are involved in regulating target genes encoding transcription factors, oxidoreductase (defense response), and UDP-glucosyltransferase, suggesting that these miRNAs possibly mediate signal transduction in response to low N by repressing translation.

Arabidopsis miR169 has been shown to be involved in adaptation to LN stress [64]. Zhao et al. [19] also reported that two novel miR169 species (miRC10 and miRC68) critically regulate the adaptation to LN in maize seedlings via their interaction with the mRNA that targets a nuclear transcription factor subunit. The strong downregulation of miR169 in Arabidopsis under LN affects the expression of its targets, NFYA (Nuclear Factor Y, subunit A) family members involved in the ABA-dependent pathway [65]. We found that two miR169s (ata-miR169d-3p_L-2R + 2 and ata-miR169d-5p) had significantly lower expression in LN-27 than in HN-27, and the predicted target gene functions included kinase activity and E3 ubiquitin-protein ligase activity. It was previously reported that Arabidopsis MIEL1 E3 ligase negatively regulates ABA signaling sensitivity by promoting MYB96 turnover [66]. Thus, the regulation of the grain response to N supply levels by miR169 might involve the ABA signaling pathway.

Previous studies have shown that miR827 responds strongly to P starvation by targeting genes encoding SPX-MFS proteins predicted to be implicated in P sensing or transport [67]. There is crosstalk between nitrate and P sensing in Arabidopsis [68], and overexpression of miR827, which targets nitrogen limitation adaption (NLA) mRNA, in Arabidopsis also resulted in similar physiological responses in both NLA and PHO2 (PHOSPHATE2-ubiquitin conjugase) mutants grown under either low nitrate or high P [68]. Fertilizer experiments also confirmed the interaction between N and P fertilizers, and the combination of N and P improved wheat grain yield and quality [69]. Here, we identified one osa-miR827, which is predicted to target a gene encoding an SPX-domain-containing protein that showed significant downregulation in LN-17. Liu et al. [70] found that NLA responded to the remobilization of nitrate between sources and sinks in Arabidopsis by regulating the degradation of NRT1.7, which depended on miR827. This finding suggests that miR827 might influence N accumulation and remobilization by regulating NLA. Additionally, the mode of action occurs via a posttranslational regulatory pathway, not at the level of transcription [71]. For all the identified differentially expressed miRNAs in our study, only one, PC-5p-26413_215, was significantly upregulated in the LN treatment at all three stages. The target gene of PC-5p-26413_215 is predicted to encode BAK1. It is well known that brassinosteroids (BRs) control various aspects of plant development and growth and that BR perception and signal transduction require active BRASSINOSTEROID INSENSITIVE 1 (BRI) and BAK1 [71]. However, additional work is needed to confirm the function of the target genes and the regulatory mechanism of this miRNA.

Nitrogen is an essential inorganic nutrient for wheat growth and plays many important roles in grain development. In this study, we found that 21 novel miRNAs were significantly up- or downregulated only in the LN-27 treatment (examples are PC-5p-6533_1330, PC-3p-15988_474, tae-miR9779-p3, PC-3p-11233_746, and tae-miR5384-p5) in comparison with HN-27, and they are predicted to target multiple genes involved in defense (peroxidase), encoding transcription factors (MYB), involved in nitrogen metabolism (metalloendopeptidase), involved in carbohydrate metabolism (UDP-glycosyltransferase), and involved in transport (hexose transport). Different expression patterns between the LN and HN treatments have also been reported by Zhao et al. [23]. Thus, the differentially expressed miRNAs and their target genes suggest that there are multiple N-responsive modules in wheat grain and that miRNAs may mediate the plant responses and adaptation to N deprivation via posttranscriptional or posttranslational regulation.

## Conclusions

We identified 79 miRNAs (46 known and 33 novel miRNAs) that showed significant differential expression during wheat grain development under both HN and LN treatments. We also identified 50 miRNAs (22 known and 28 novel miRNAs) that responded to the level of applied nitrogen. The potential targets of these miRNAs are involved in multiple biological processes, including cell differentiation, carbohydrate metabolism, transcription, signal transduction, transport, and defense. Our results indicate that posttranscriptional regulation by miRNA-mediated networks can play a crucial role in grain development and the N response, which determine wheat grain weight and quality. Our work provides useful information for further exploration of the mechanisms by which miRNAs function to improve grain yield and quality.

## Methods

### Experimental design and sample preparation

Seeds of the winter wheat cultivar ‘ZM119’, provided by Henan Academy of Agricultural Sciences, were grown during the 2016–2017 growing season at the Scientific and Educational Park of Henan Agricultural University, Yuanyang, China (35°06′ N, 113°56′ E). The cultivar ‘ZM119’ was bred by Henan Academy of Agricultural Sciences, and approved by Henan Province Crop Variety Approval Committee in 2014. The organic matter content in the topsoil was 10.1 g kg^− 1^, and the available phosphorus and available potassium contents were 35.0 mg kg^− 1^ and 110.6 mg kg^− 1^, respectively. Two N fertilizer treatments with three replicates each were applied: LN (0 kg ha^− 1^) and HN (210 kg ha^− 1^). The area of each plot was 21 m^2^ (3 m × 7 m), and each plot received 315 g of P (P_2_O_5_) and 315 g of K (K_2_O) before sowing. Fifty percent of the total N (in the form of urea) was applied before sowing, and 50% was topdressed at the elongation stage. Seeds were planted on 14 October 2016 at a density of 200 seeds m^−2^. At flowering, wheat spikes having a similar size and flowering on the same day were marked. The wheat spikes were sampled at 7, 17, and 27 DAA. The sampled grain was immediately frozen in liquid nitrogen and then stored in an ultra-low-temperature refrigerator until use. Afterward, mature grain was ground in a Cyclotec Sample Mill (Foss Tecator AB, Höganäs, Sweden) after cleaning and removing the foreign grain.

### Small RNA library construction and RNA sequencing

To construct eighteen sRNA libraries, six independent samples, HN-7, HN-17, and HN-27 as well as LN-7, LN-17, and LN-27 (grain sampled at 7, 17, and 27 DAA under HN and LN conditions, respectively) with three biological replicates were separately extracted using TRIzol reagent (Invitrogen, Carlsbad, CA, USA) according to the manufacturer’s instructions. The RNA quantity and quality were assessed using a Bioanalyzer 2100 and an RNA 6000 Nano LabChip Kit (Agilent Technologies, Santa Clara, CA, USA). RNA preparations with a RIN (RNA integrity number) > 7.0 were used for sRNA library construction. Small RNAs were first ligated to 5′ RNA/DNA chimeric oligonucleotide adaptors (Illumina) and then 3′ adaptor to generate ligation products. cDNAs were then produced by reverse transcribing the ligation products. The cDNAs were sequenced using an Illumina HiSeq 2500 instrument (Illumina, San Diego, CA, USA) according to the manufacturer’s recommended protocol.

### Bioinformatic analysis and miRNA identification

The raw sequencing reads in the miRNA libraries were subjected to an Illumina pipeline filter (Solexa 0.3), and then an in-house program, ACGT101-miR (LC Sciences, Houston, TX, United States), was used to process the dataset for removing adaptor dimers, common RNA family members, repeats and low-complexity reads. Unique sRNA sequences between 18 and 25 nucleotides in length were subsequently mapped to specific species precursors in miRBase 21.0 (http://www.mirbase.org) using BLAST searches to identify conserved miRNAs and novel 5p- and 3p-derived miRNAs [72]. Internal mismatches in the sequence and length variations at the 3′ and 5′ ends were allowed in the alignment process. The unique sequences that mapped to specific mature miRNA species in the hairpin arms were characterized as known miRNAs. A potential novel 5p- or 3p-derived miRNA was identified when unique sequences were mapped to the other arm of known specific species of precursor hairpins, opposite to the annotated mature miRNA arm. The remaining sequences were further matched to other selected species precursors in miRBase 21.0 by BLAST searches, and the mapped pre-miRNAs were further used as BLAST queries to identify their genomic locations by searching the genomes of specific species. The results of the above two searches were classified as known miRNAs. Finally, unmapped sequences were queried via BLAST against the specific genomes, and hairpin RNA structures containing the sequences from the flanking 120-nt sequences were predicted using RNAfold software (http://rna.tbi.univie.ac. at/cgi-bin/RNAfold.cgi). The key criteria for miRNA prediction are reported in the literature [73]. The secondary structures of precursors were also predicted using RNAfold (http://rna.tbi.univie.ac.at/cgi-bin/RNAWebSuite/RNAfold.cgi). The raw data have been submitted to the Sequence Read Archive (SRA) at the NCBI (https://www.ncbi.nlm.nih.gov) under accession number PRJNA563099.

### Identification of differentially expressed miRNAs

The expression levels were normalized as RPM values. A *t*-test was applied to evaluate statistical significance. If the RPM ratios between different treatments were greater than 2 (fold change ≥ 2) and if the *P*-value was ≤0.05, then the miRNAs were defined as differentially expressed miRNAs.

### miRNA target prediction and annotation

The genes targeted by miRNAs were predicted by identifying miRNA-binding sites using computational target prediction algorithms (Target Finder). Target searching was performed using the *Triticum aestivum* L. IWGSC library (http://plants.ensembl.org/index.html). Potential miRNA targets were functionally annotated by querying the target sequence via BLAST against the NCBI library (https://www.ncbi.nlm.nih.gov/). The most abundant miRNA targets were also annotated with GO terms (http://www.geneontology.org/) and KEGG pathways (http://www.genome.jp/kegg/).

### miRNA and target mRNA quantification using qRT-PCR

Reverse transcription reactions were carried out using miRNA First-Strand cDNA Synthesis SuperMix (TransScript) according to the manufacturer’s instructions. A SYBR PrimeScript miRNA RT-PCR Kit was used to perform qRT-PCR in a fluorescence detection system (TianGen Biotech, Beijing, China) following the manufacturer’s instructions. All reactions were performed in triplicate for each sample. The 2^-∆∆CT^ method was then used to calculate the relative expression levels of the miRNAs [74]. Target genes of the miRNAs that were differentially expressed during grain development in the HN and LN treatments were selected to validate their expression patterns in developing wheat grain via qRT-PCR. Fisher’s least significant difference test (LSD) was subsequently used to distinguish differences in relative expression levels between different development stages using SPSS (Statistical Program for Social Science) software; *P* < 0.05 was considered statistically significant. All primer sequences used and the target gene identification results are given in Table S7 (Additional file 8).

### Determination of grain total protein content and the individual fraction contents

Four grain protein fractions, albumins, globulins, gliadins, and glutenins, were extracted using the sequential extraction methods of Liu et al. [75]. The total protein content and the individual fraction contents in each sample were analyzed by an automatic Kjeldahl analyzer (Kjeltec 2300, Foss Tecator, Sweden) following the method of ICC (1994) Standard Method 105/2 (International Association for Cereal Chemistry, Vienna, Austria). Differences between HN and LN were evaluated with *t*-test via SPSS; A value of *P* < 0.05 was considered to be statistically significant.

## Supplementary information

**Additional file 1: Table S1.** Illumina DNA sequencing data for the small RNA libraries.

**Additional file 2: Table S2.** miRNAs identified in this study.

**Additional file 3: Table S3.** Novel miRNAs identified in developing wheat grains at two different N levels.

**Additional file 4: Table S4.** Conserved and novel miRNAs that are differentially expressed during wheat grain development.

**Additional file 5: Table S5.** Target function of differentially expressed miRNAs during grain filling under HN and LN treatments.

**Additional file 6: Table S6.** Wheat grain miRNAs that are differentially expressed between the HN and LN treatments.

**Additional file 7: Figure S1.** Relationships between differentially-expressed miRNAs identified between the HN and LN treatments and their predicted targets as determined by Cytoscape.

**Additional file 8: Table S7.** Oligonucleotide primers used for qRT-PCR assays in this study.

## Data Availability

All data generated or analyzed during this study are included in this published article (and its supplementary information files). The sequencing raw reads were submitted to the NCBI database with the accession number PRJNA563099 (https://www.ncbi.nlm.nih.gov).

## Abbreviations

ABA: Abscisic acid
ARF: Auxin response factor
BAK1: BRASSINOSTEROID INSENSITIVE 1-associated receptor kinase 1
BRI: BRASSINOSTEROID INSENSITIVE 1
CRF: Cytokinin response factor
DAA: Days after anthesis
GA: Gibberellin
GO: Gene ontology
GSTF: Glutathione S-transferase
HN: High nitrogen treatment
HSP: Heat-shock protein
LN: Low nitrogen treatment
LRR: Leucine-rich repeat
miRNA: microRNA
N: Nitrogen
NLA: Nitrogen limitation adaption
qRT-PCR: Quantitative reverse transcription-polymerase chain reaction
SCF: Skp-cullin-F-box; SPXSYK1/PHO81/XPR1
sRNA: Small RNA
