# Supplementary figures and images for: Identification of microRNAs in developing wheat grain that are potentially involved in regulating grain characteristics and the response to nitrogen levels
Source: BMC Plant Biol. 2020 Feb 27;20:87. doi: 10.1186/s12870-020-2296-7 (PMC7045451; doi:10.1186/s12870-020-2296-7)

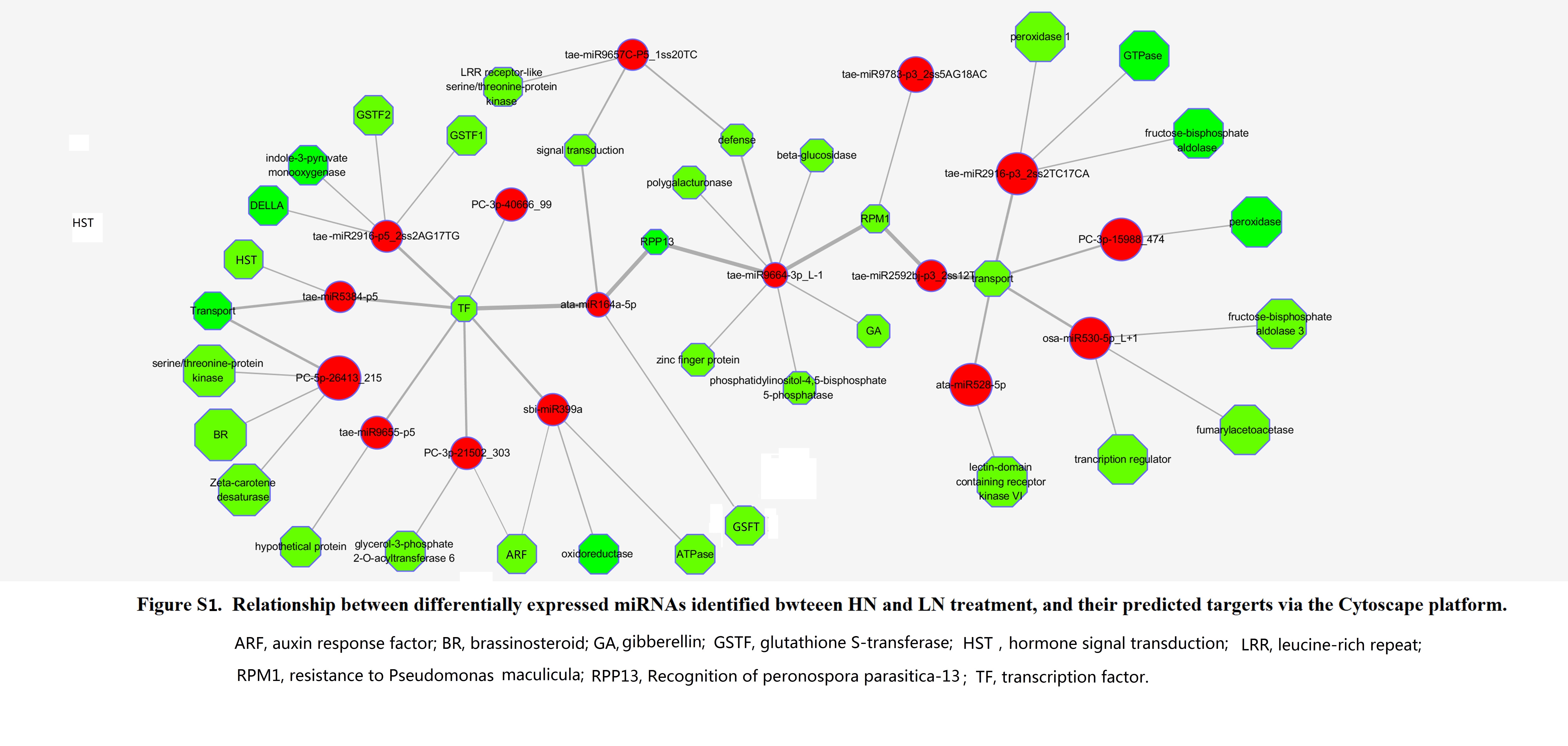

Supplement: Supplementary file 7 — Additional file 7: Figure S1. Relationships between differentially-expressed miRNAs identified between the HN and LN treatments and their predicted targets as determined by Cytoscape. [file 12870_2020_2296_MOESM7_ESM.jpg]
